# Two distance memories in desert ants—Modes of interaction

**DOI:** 10.1371/journal.pone.0204664

**Published:** 2018-10-10

**Authors:** Harald Wolf, Matthias Wittlinger, Sarah E. Pfeffer

**Affiliations:** 1 Institute for Neurobiology, University of Ulm, Ulm, Germany; 2 Institute of Biology I, Neurobiology and Behaviour, University of Freiburg, Freiburg, Germany; Universitat Bielefeld, GERMANY

## Abstract

Navigation plays an essential role for many animals leading a mobile mode of life, and for central place foragers in particular. One important prerequisite for navigation is the ability to estimate distances covered during locomotion. It has been shown that *Cataglyphis* desert ants, well-established model organisms in insect navigation, use two odometer mechanisms, namely, stride and optic flow integration. Although both mechanisms are well established, their mode of interaction to build one odometer output remains enigmatic. We tackle this problem by selectively covering the ventral eye parts in *Cataglyphis fortis* foragers, the eye regions responsible for optic flow input in odometry. Exclusion of optic flow cues was implemented during different sections of outbound and inbound travel. This demonstrated that the two odometers have separate distance memories that interact in determining homing distance. Possible interpretations posit that the two odometer memories (i) take on different relative weights according to context or (ii) compete in a winner-take-all mode. Explanatory values and implications of such interpretations are discussed. We are able to provide a rough quantitative assessment of odometer cue interaction. An understanding of the interaction of different odometer mechanisms appears valuable not only for animal navigation research but may inform discussions on sensor fusion in both behavioural contexts and potential technical applications.

## Introduction

*Cataglyphis* desert ants are accomplished navigators in the barren landscape of North African salt pans, dunes and steppe biotopes [1, 2]. During foraging trips, ants may stray from their nest for distances exceeding 10.000 body lengths [3, 4]. *Cataglyphis* has thus become a well-studied model organism in animal navigation research [3, 5]. The ants’ major means of orientation is path integration, combining angles steered and distances travelled during outbound travel into a home vector that guides them back to their nest. To estimate covered distances, *Cataglyphis* employs two odometer mechanisms [6], namely, stride and optic flow integration [7–9], that appear to represent two independently evolved odometers [9, 10]. Both mechanisms are well established but their mode of interaction to control homing behaviour remains enigmatic. Indeed, the interaction of odometer mechanisms has not been tackled before, contrasting with the interactions of different compass cues [11–14] and compass and landmark cues [14–17] that have been addressed previously.

In the present study, we examine interaction of the two odometers and their respective memories by the transient inactivation of one of the two mechanisms. The stride integrator is invariably active in walking ants [18] and thus cannot be disabled in *Cataglyphis* foragers. We therefore eliminated optic flow perception by covering the ventral eye parts in foraging desert ants. These eye regions are responsible for optic flow input in odometry [7, 9]. The exclusion of optic flow cues was implemented during different sections of outbound and inbound travel to assess the relative contribution of the optic flow odometer. This demonstrated that stride and optic flow odometers have independent memories that interact in determining homing distance. Interaction of the two odometer memories may be conceptualised by a set of (related) interpretations. One straightforward interpretation assumes that the two odometer memories together drive homing with relative weights that vary according to context. The present experiments provided a rough quantitative assessment of these respective weights.

The experiments reported below often follow a logic where the design of a given experiment is based on the outcome of the previous experiment. We therefore outline a set of working hypotheses related to the experimental design and previous reports in the literature. This is meant to facilitate understanding of the rationale of the experimental sequence, while the Discussion will pick up these hypotheses, scrutinize them, and put them in the context of possible interpretations.

1. **Optic flow and stride integrator odometers have separate distance memories, and these are charged in parallel**. In previous reports, separate odometer memories have either been strongly suggested in *Cataglyphis cursor* [19] and have actually been demonstrated in *Cataglyphis bicolor* [9].
2. **The two odometer memories interact in driving homebound travel with adjustable relative weights**. Together with hypothesis 1 this implies that one odometer alone should be able to guide the ant towards the nest in particular situations, e.g. with one odometer is incapacitated, while under normal conditions both are interacting to establish odometer output.
3. **The memory contents of an odometer is maintained at its current value if the odometer is** (temporarily) **incapacitated**. That is, transient exclusion of one odometer results in maintenance of its memory contents that can be re-activated later.
4. **The optic flow and stride integration memories are discharged separately**. The two distance memories can be charged, as postulated by hypothesis 1, and also discharged independently.
5. **A nest search is initiated as soon as one of the odometer memories has been depleted, and the other odometer memory has either also been discharged to zero, or it is inactive and cannot be used**. This hypothesis has indeed been verified previously in different contexts. One example are local vectors that are activated by landmark arrays [20], and that may drive homing behaviour even when the path integrator has been run off, signalling the nest position, and *vice versa*.

## Material and methods

### 1. Animals and experimental situation

Experiments were performed on a field site near Maharès (34° 31’ 46” north, 10° 32’ 24” east), a Tunisian village just south of Sfax. The experimental season lasted from the end of May until the beginning of September in 2007 to 2009 and in 2015 to 2016. Large and active nests of *Cataglyphis fortis* (Forel 1902) [21] were selected for the experiments. The experiments reported here comply with all requirements concerning animal welfare and are in full accord with current laws in Germany and Tunisia. No endangered or protected species were involved in our field studies. The research conducted was covered by a research permission of the national authorities (Ministère des eaux et forêts) and kindly approved by the owner of the land.

### 2. Procedures for behavioral experiments

#### 2.1 Eye covers

It was the objective of the present experiments to study the mode of interaction of two odometer memories, namely, stride integrator [8, 22] and optic flow integrator memories [7, 9]. Ants were therefore trained and tested with or without ventral eye covers that eliminated visual input from the ommatidia of the ventral half of the compound eyes. The ventral parts of the eyes were painted over with car paint (Motip Dupli GmbH, Haßmersheim, Germany), sometimes already on the day before testing. There was no difference in the behavior of ants that had their eye covers applied immediately before and on the day before the respective experiment. That is, there were no effects of longer lasting deprivation of ventral eye input. The eye covers were checked with a hand held 10x magnifying glass after the paint had set, and they were re-checked before every test run. In some experiments, the ventral eye covers were removed in the course of the test runs, or they were (re-) applied during the test procedure. In these cases, too, successful removal and (re-) application were checked with the magnifying lens.

#### 2.2 Channel set-up and feeder

An ant nest was connected to a feeder established in 10 m distance by a linear U-shaped aluminium channel (training channel). In addition, a channel aligned in parallel and 35 m long (test channel) was installed at a short distance from the training channel. The profiles of training and test channels were 7 cm wide and the walls 7 cm high. This provided the ants with a strip-like view of the sky of approximately 53° to read the skylight pattern and derive compass information. The channel walls were painted homogeneously with dull grey varnish to eliminate reflections, potential landmark cues, and lateral optic flow cues [23]. The channel floor was coated with fine grey sand to provide an even ground with traction for walking and homogeneous ventral optic flow. The feeder in the training channel was equipped with cookie crumbs cut and sieved to a size of approximately 1.5 by 1.5 mm to produce food items easily accepted by the ants. A few drops of peach juice made these morsels even more attractive.

#### 2.3 Training and test procedures

**Fig 1:**
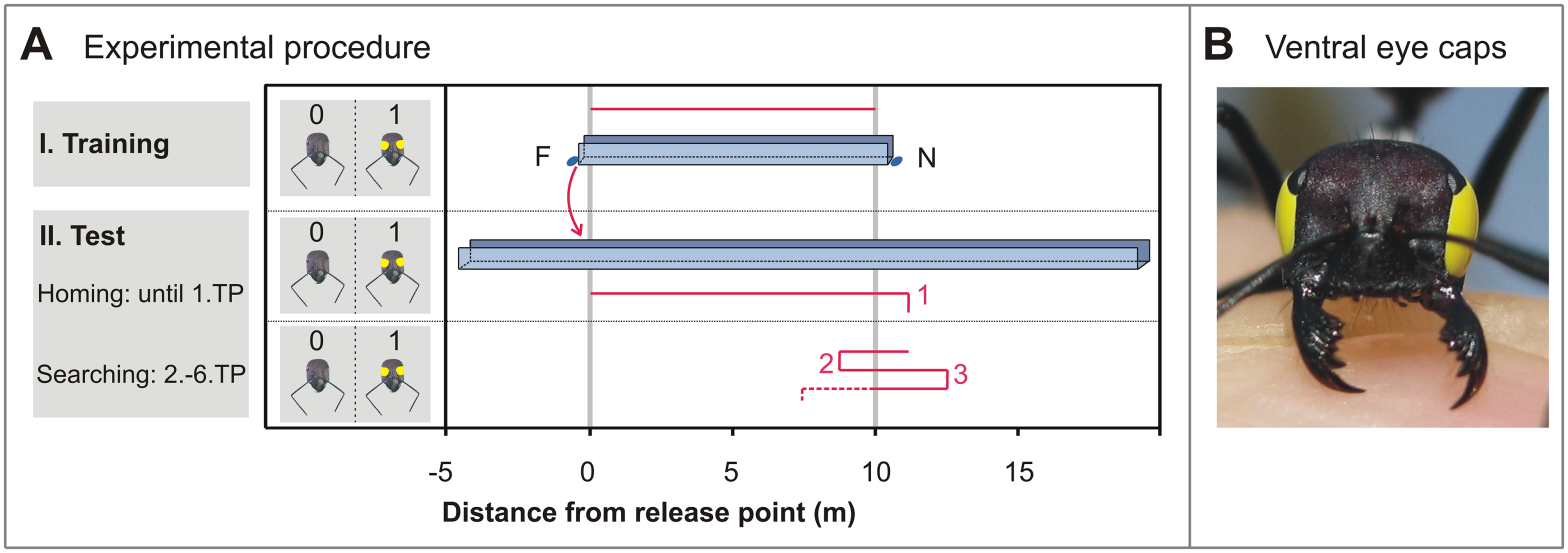
Examining ant homing behaviour. A) During I. Training (outbound), a nest (N) was connected via an aluminium channel to a feeding station (F) at 10 m distance. After arriving at the feeder, an ant forager was captured and transferred to a distant channel, where it performed its inbound journey and thus completed the experiment (II. Test). The ant’s homebound travel and consecutive turning points (1, 2, 3…, not drawn to scale) during searching were noted to assess homing performance. (B) During different stages of an experiment (Training, Homing until 1. turning point (TP), Homing from 2. to 6. TP), exclusion of optic flow cues was achieved by ventral eye caps. Presence (1) or absence (0) of eye caps is indicated in insets left to the channel drawings. 0–10, for example, signifies the absence, presence and again absence of eye caps during training, homing until the 1. TP, and searching from the 2. to 6. TPs, respectively; 0–00 signifies the sham controls without eye caps at any stage.

Experiments were performed according to established procedures [22, 24]. Individually marked ants had visited the feeder at least five times in the training channel before experiments were started. Ants that had taken up a food crumb were gently caught at the feeder and transferred to the test channel to record their homing performance. Only those ants were considered that held on to their food crumb for the entire homebound trajectory, even in the cases of interruptions of homing behavior for removal or (re-)application of eye covers. Transport of a food item indicates that an ant is motivated to carry the morsel back to the nest and is thus indeed on its homebound journey [25]. Once placed in the test channel, the animals took up determined homebound runs. A switch from homing behavior to nest searching (compare [26]) is indicated by a conspicuous U-turn, followed by a search pacing back and forth around the assumed position of the nest entrance [27]. We recorded the point of this switch in homing behavior (first turning point, 1. TP), as well as six consecutive turning points of the subsequent search. U-turns were recorded only when the new walking direction was maintained for at least 40 cm before another turn occurred (Fig 1).

Depending on the experimental paradigm (see below), the ants’ ventral eye halves were manipulated during three different experimental phases: (i) during outbound training, (ii) until the first turning point (1. TP) of homing and (iii) during the second to sixth turning points (2.-6. TP).

#### 2.4 Experimental paradigms

The following training and test paradigms were carried out, with “0” denoting intact ants without eye covers, and “1” meaning that the ventral eye portions had been painted over. In the labels used below (e.g. 0–00), the hyphen (—) separates training and test runs. In some experimental series, eye covers were changed–removed or re-applied–during the test phase, after the first turning point, which is indicated by the two figures behind the hyphen.

1. 0–00: One group of control ants (sham control) was trained and tested without eye covers. These ants were caught at the feeder and their ventral eye fields were painted over for 30s. Afterwards, the eye caps were removed again and the ants were released into the test channel to record their homebound walking trajectories and six consecutive turning points of nest search behavior.
2. 1–11: Another control group was trained and tested with eye covers present throughout the experiment. Again, we recorded the homebound trajectory and six consecutive turning points.
3. 0–11: Ants were trained with intact eyes and subsequently tested with eye covers. Again, homing and six consecutive turning points were recorded.
4. 0–10: Ants were trained with intact eyes, ventral eye covers were applied for testing until the animals had exhibited the first turn and thus initiated their nest search; the animals were captured within 20 cm of the turning point, the eye covers were removed, and the ants released at the point of capture to record another five turning points.
5. 1–00: Ants were trained with ventral eye covers present and the covers were removed for testing. We recorded six consecutive turning points.
6. 1–01: Finally, ants trained with ventral eye covers had the covers removed to record the first turning point. The animals were captured within 20 cm of the turning point and the eye covers were re-applied before recording the next five turns of homing behavior.

### 3. Analysis of homing performance

As described above, we recorded six consecutive turning points to obtain representations of the ants’ searching performance and homing distance (as the center of the search, or the first turning point). To do so, the test channel was divided into 10 cm bins by stringing a tape measure along the channel, and the turning points were noted with regard to these bins for further analysis.

**Fig 2:**
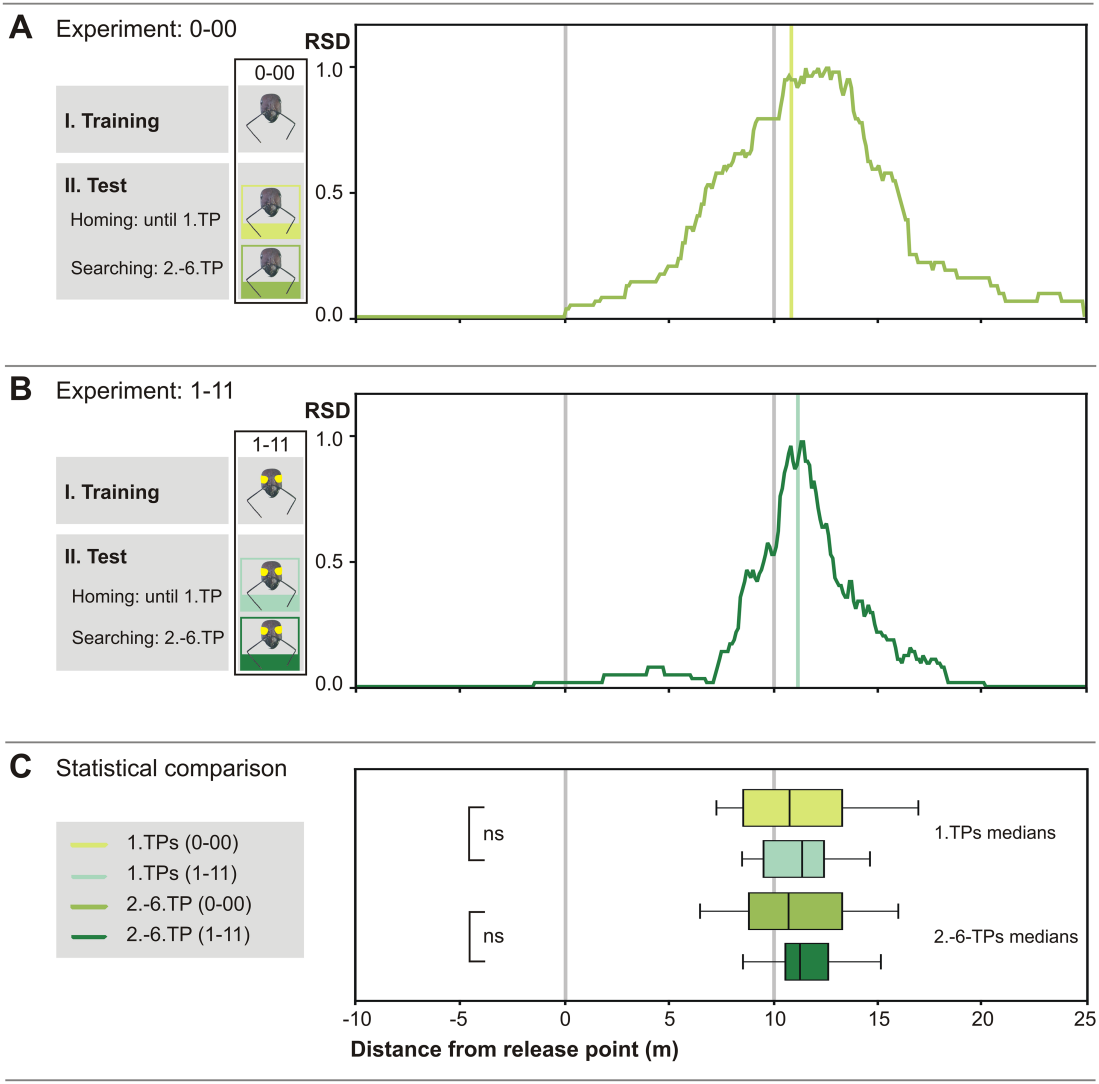
Correct distance measurement is possible without optic flow cues. (A) Median of 1. TPs and relative search density (RSD) plot of 0-00-experiment (n = 26). Ants of this sham control group were able to perceive optic flow during the entire experimental procedure. However, between I. Training and II. Test, eye caps were applied and removed 30s afterwards. (B) Median of 1. TPs and relative search density (RSD) plot of 1-11-experiment (n = 26). Ants were deprived of ventral optic flow cues during the entire experimental procedure. Release point (0) and fictive nest position (10) are marked by grey vertical lines, and 1. TP medians by coloured lines, in this and the following figures. (C) Box-and-whisker plots and statistical analysis. There is no statistically significant difference between the grand medians of the 0–00 and the 1–11 groups, demonstrating correct distance estimation for both groups. Nevertheless, the RSD-plot reveals a narrower search distribution of the 1-11-experiment, the reason remaining unclear.

**Fig 3:**
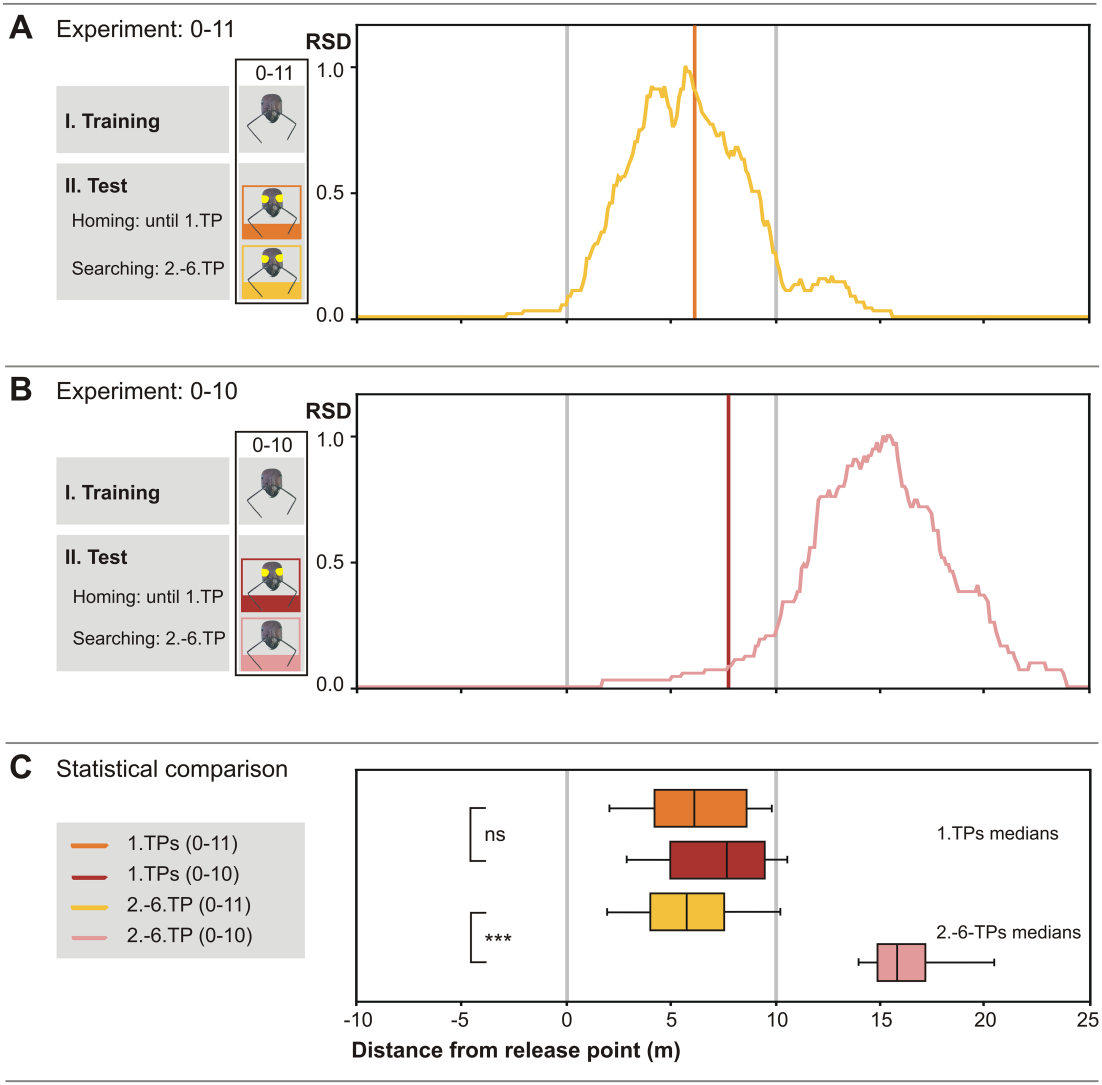
Stride and optic flow odometer memories interact. (A) Median of 1. TPs and relative search density (RSD) plot of 0-11-experiment (n = 39). Ants approached the feeder without eye covers but were deprived of optic flow cues during the entire homing run. (B) Corresponding plot of 0-10-experiment (n = 26). Ants were deprived of optic flow cues during homing until they had exhibited their 1. TP, where eye covers were removed. (C) Box-and-whisker plots and statistical analysis. There is no statistically significant difference between the 0–11 and the 0–10 groups regarding the 1. TPs (p = 0.329, t-test). However, there is a significant difference between the search densities exhibited by the 0–10 and 0–11 groups (p<0.001, U-test). That is, restoring normal optic flow perception after the 1. TP results in the ants resuming homebound travel over appreciable distance.

**Fig 4:**
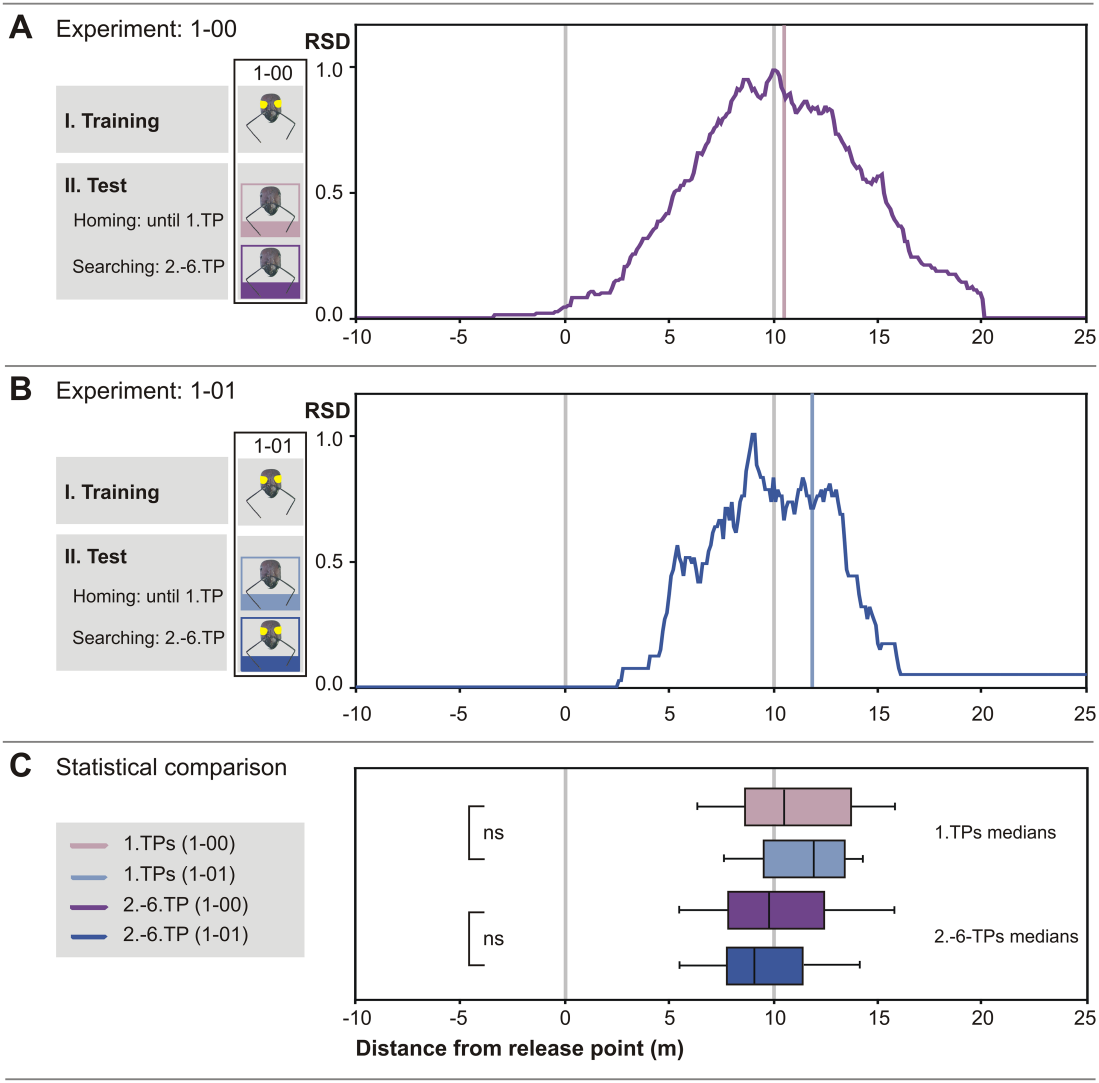
An odometer mechanism that is inactive at the time of nest exit does not contribute to homing, even if activated later on. (A) Median of 1. TPs and relative search density (RSD) plot of 1-00-experiment (n = 91). Ants were deprived of optic flow during the entire outbound run and eye covers were removed for the homebound journey. (B) Corresponding plot of 1-01-experiment (n = 30). Ants were deprived of optic flow cues during the outbound run, eye covers were removed before the start of the homebound run and re-applied after an ant had exhibited its 1. TP. (C) Box-and-whisker plots and statistical analysis. There is no statistically significant difference between the 1–00 and the 1–01 groups regarding 1. TPs (p = 0.358, U-test) and grand medians of the subsequent search densities (p = 0.637, t-test). Differences in optic flow input between outbound and inbound travel *per se* thus do not cause misjudgement of walking distance.

First, search density distributions were evaluated for each experimental situation. This analysis illustrates how often an ant has visited a certain bin of the test channel during its six consecutive turns. These visits were counted and cumulated to calculate the relative distribution. This means, the more often a channel segment (10 cm bin) was visited during the search, the higher was its value in the density distribution. Search density distributions were normalized to their peak values for presentation in the figures (Figs 2–4, S1 Fig).

Second, the median values of the turning points of the ants’ nest searches were calculated to provide an estimate of the search center. From these data, box-and-whisker plots were constructed. Marked values in the box-and-whisker plots are the center (grand median), the spread (inter-quartile range, IQR, 25% and 75% percentiles), and the 10th and 90th percentiles (whiskers) (Figs 2–4, S1 Fig).

### 4. Statistical analyses

We determined medians instead of means because not all data were normally distributed. Pair-wise comparisons were performed between relevant experimental groups. The t-test was used for normally distributed data and the Mann–Whitney rank sum test (U-test) for non-normally distributed data. A Bonferroni correction was applied considering the multiple use of our control group for comparison, and we thus tested for a significance level of p = 0.01, instead of 0.05. Sample sizes ranged from n = 20 to n = 91. All experimental groups consisted of different sets of animals and thus were independent. Tests were performed with SigmaPlot 11.0 (Systat Software, San Jose, CA, USA).

## Results

### Examining ant homing behavior; sham (handling) controls

Ants are accomplished navigators using a multitude of cues for orientation (e.g. [14]). To focus our study on odometer function we performed experiments in straight channels, thus excluding other orientation cues such as panorama (e.g. [28]) and landmarks (e.g. [29]). The ants completed their outbound journey in a training channel connecting the nest to a feeding station. Before starting their homebound travel, ants were gently captured and transferred to a parallel test channel, where homing and subsequent nest search were recorded (Fig 1A). By covering the ventral parts of the compound eyes with light-tight paint we excluded optic flow input during different sections of the ants’ foraging trips (Fig 1B).

Animals of a sham control group (0–00) (terminology see legend Fig 1 and Methods), were caught at the feeder, at 10 m distance from the nest, and had ventral eye covers applied that were removed again after just 30s. Afterwards, they were transferred to the test channel to record their typical homing and nest search behaviours (Figs 1A and 2A). One measure of an ants’ distance estimate is the point where the nest search is initiated, that is, the first turning point (1. TP) after a fairly straight and uninterrupted homebound run [7, 30, 31]. The median of the first turning points of the 26 control ants was at 10.80 m (interquartile range, IQR 8.60 m to 13.10 m). Another measure of an ant’s distance estimate is the search centre, determined as the median of the subsequent second to sixth turning points (2.-6. TP). In the present experiment, the grand median (median of all individuals’ search medians) of the nest searches was at 10.75 m (IQR from 8.90 m to 13.60 m), just 75 cm past the correct nest-feeder distance.

Our sham (handling) control group is thus indistinguishable from the normal search behaviour of untreated ants in channel arrangements (1. TP median 11.00 m, search median 10.60 m; see also [22]). Apparently, neither handling nor eye painting procedures influenced the ants’ search behaviour (see S1 Fig).

### Homing performance of ants with ventral eye covers

In this experiment, we covered the ants’ ventral eye halves during both outbound (training) and inbound (testing) journeys (1–11; see legend Fig 1 and Methods for terminology). These animals used their stride integrator but had no ventral optic flow available to measure walking distance [7, 29]. Ventrally blinded ants gauged travel distance correctly (Fig 2B), and their homing performance was statistically indistinguishable from the sham control group (Fig 2C) (1. TP median 11.40 m (IQR 9.63 m to 12.43 m; p = 0.544, U-test), search distance grand median 11.30 m (IQR 10.65 m to 12.53 m; p = 0.371, U-test)). The fact that the search is close to the 10 m mark demonstrates that optic flow cues are not essential for an ant to measure walking distance rather correctly in the present situation, in agreement with previous studies [19].

Optic flow was absent during the whole foraging trip in these experiments (1–11 and [19]). Exclusion of optic flow during the homebound journey only, that is, with charged optic flow memory according to the above hypotheses, has not yet been examined. This particular experiment (0–11) should however allow to ascertain if the two odometer mechanisms indeed operate independently and how they interact in determining homing distance.

### Exclusion of optic flow cues during the homebound journey only

To address the interaction of optic flow and stride integration, we first applied eye covers only during the homebound journey. That is, the ants were captured at the feeder as usual but before releasing them into the test channel their ventral eye halves were painted over (0–11). These animals consistently searched for the nest at remarkably shortened distances, well below 10 m (Fig 3A and 3C). In the present experiment, the median search distance (grand median) of the experimental ants was at 5.70 m (IQR 4.03 m to 7.40 m) and the median position of the first turning points was at 6.10 m (IQR 4.20 m to 8.55 m). The difference between the search distances of this experimental group (0–11) and the handling control animals (0–00) was significant (1. TPs, p<0.001, t-test; 2.-6. TPs, p<0.001, U-test; both significant after Bonferroni correction).

According to the hypotheses outlined in the Introduction, this distinct–and from a naïve perspective, unexpected—shortening of homing distance may be a result of the charged optic flow memory that has been (temporarily) incapacitated by the eye covers. Testing whether or not an optic flow memory is actually present in the ants that have reached the nest according to their stride integrator is thus the next logical experiment.

### Exclusion and subsequent re-activation of optic flow during homebound travel

This experiment was carried out by applying eye covers before an experimental ant started its homebound travel as before, and removing the eye covers once the animal had commenced its nest search, indicative of the stride integrator memory having been run off. The results of this experiment (0–10) are shown in Fig 3B and 3C. After removal of the eye caps at the first turning point (median 7.65 m; IQR 5.10m to 9.40m), the ants travelled further into the nest direction, actually overshooting the fictive nest position. The second nest search commenced at a median distance of 16.10 m (IQR 15.60 m to 17.80 m; median of 2. TPs; not shown in figures) from the initial release point (i.e. fictive feeder position), and the search median was at 15.80 m (IQR 14.90 m to 17.00 m). When comparing the search medians in the 0–11 and 0–10 experiments, the difference is 10.10 m, a value close to the assumed 10 m content of the optic flow integrator when removing the eye caps after the first turning point. When considering homing distance (search grand median) with respect to the second release point (i.e. the point of eye cap removal at the 1. TP, median 7.65 m), instead of the original release point (0 m), the ants covered a median distance of 8.15 m.

### Exclusion of optic flow cues during the outbound journey

The preceding set of experiments requires completion by the remaining combinations of eye cover applications to address the hypotheses outlined in the Introduction and potential explanations for the above results. For example, a change of ventral eye covers might affect homing performance in itself. In addition to the 1–11 and 0–00 experiments reported above, we therefore performed the 1–00 and 1–01 experiments (i.e. the reverse of the 0–11 and 0–10 experiments above).

The first turns recorded after removal of the eye covers just after transfer to the test channel had median distances of 10.50 m (IQR 8.60 m to 13.68 m) for the 1–00 experiment, and of 11.95 m (IQR 9.60 m to 13.30 m) for the 1–01 experiment. Similar results were obtained when examining search behavior after the first turning point (2.-6. TP). In the 1–00 experiment, the search grand median was at 9.80 m in this case. Re-applying the ventral eye covers in the 1–01 experiment produced little change in the search center, too, with the search median at 9.05 m (IQR 7.80 m to 11.10 m) (Fig 4) and without any statistically significant difference to the sham control group (1. TPs, p = 0.824, t-test and 2.-6. TPs, p = 0.052, U-test; both not significant also after Bonferroni correction).

These results demonstrate that a change of ventral eye covers in itself does not affect homing performance, and neither does the absence of optic flow input during the outbound journey, as shown previously (1–11 experiment, [19]).

## Discussion

### Interpreting the experimental results in context

**Fig 5:**
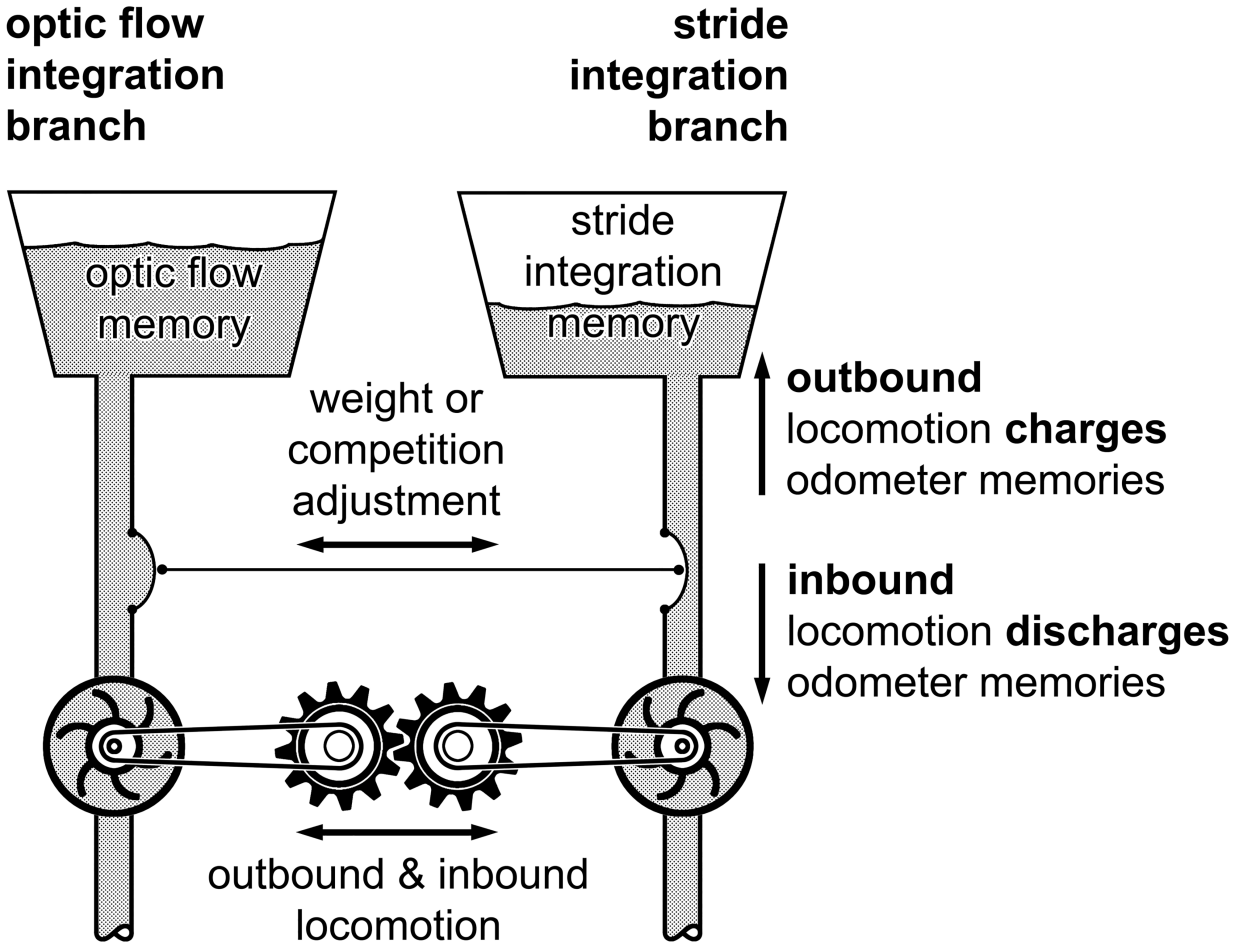
Hydraulic model of optic flow integration (left half of diagram) and stride integration (right half) odometry in *Cataglyphis* ants. The diagram illustrates the idea of separate optic flow and stride integration memories and their possible interaction. Depiction as hydraulic model was already put forward by Konrad Lorenz’ psycho-hydraulic model of instinct behaviour [52, 53]. Outbound and inbound locomotion (symbolised by cogwheels, bottom centre) drive the optic flow and stride integrators (turbine pumps, driven by cogwheels). The distance integrators fill or deplete their respective distance memories (elevated water troughs above turbine pumps) during outbound or inbound travel, respectively (arrows on the right). Either odometer may work alone when the other one is incapacitated (cogwheels would be disengaged, only one of them active). Normally, however, the two odometer memories drive homing together, although with different weights, or competitively. This is illustrated by two interconnected membrane valves that open or close the supply pipes to the water troughs at each other’s expense. Competitive interaction is indicated by the valve to the trough with the higher water level, and thus higher water pressure, constricting the valve in the pipe with the lower pressure. Although both odometers would normally measure the same walking distance, the (symbolic) water levels need not to be identical. Neither the denomination nor even the currency of the two odometer memories have to be identical (see Discussion, potential neuronal substrates of odometers).

Before scrutinising the hypotheses listed in the Introduction in detail, a possible interpretation of the above results shall be presented. This conceptualisation is intended to illustrate our general lines of thought, and thus facilitate subsequent discussion of critical points of this interpretation later on. We further provide an illustrative summary of possible odometer interaction in the form of a hydraulic model (Fig 5).

In the normal life of an ant (0–00), the stride and optic flow odometers both measure walking distance. In the present experiments this results in a value of 10 m in each of the odometer memories *(****hypothesis 1***, *parallel charging of separate odometer memories)*. In homing, the two odometers interact in driving homebound travel, each with a particular relative weight (“relative” is meant to indicate that the total is 1.0 under normal circumstances). These weights are adjusted according to the behavioural situation (see below) *(****hypothesis 2***, *interaction with different relative weights of the two odometers)*. In the 1–11 experiment, for example, the optic flow integrator remains “empty” from the outset and cannot contribute to homing. The relative weight of the stride integrator is thus 1.0. In the 0–11 experiment, by contrast, the homing distance is shortened to about 6 m, and thus the stride integrator must have assumed a relative weight of about 0.6 (and the optic flow integrator, 0.4). The ventral eyes covers incapacitate the optic flow integrator and only the stride integrator is able to drive homing–much like in the 1–11 experiment. However, the optic flow integrator has been charged during outbound travel to 10 m, and this charged optic flow memory appears to have set the stride integrator’s weight to a value normally appropriate for homing. The memory of the optic flow integrator maintains this value since it cannot be read out with eye covers present *(****hypothesis 3***, *odometer memory is maintained at its current value if not active)*. Once the stride integrator is depleted, a nest search is initiated (despite the still charged optic flow memory!) *(****hypothesis 5***, *nest search is initiated as soon as one of the odometer memories has been depleted*, *and the other odometer memory is either also zero*, *or the odometer is inactive and cannot be used)*. When removing the eye covers at the point where the first nest search has been initiated, the ants resume homebound travel, driven by the still charged optic flow integrator *(****hypothesis 4***, *separate discharging of the two distance memories)*. A quantitative assessment of the result of the 0–10 experiment is provided further below.

The above hypotheses appear consistent, are in agreement with the above results, and together correctly predict the outcome of the (critical) 0–10 experiment. Nonetheless, a set of other interpretations exists, of course. This concerns in particular the mode of interaction of the odometer memories. For example, instead of assigning different (complementary) weights to the two memories in driving homebound travel, one may postulate a competitive interaction (*modified hypothesis 2*). An appropriately adjusted (soft) winner-takes-all scenario (compare [17]) is in agreement with the present results, too. The odometer memory with the higher charge level reduces contribution of the competing memory to homing performance (compare Fig 5)–perhaps biased towards stride integration that appears to take precedence in normal foraging situations (compare [7]). This may be achieved, for instance, by inhibiting the charging and promoting the depletion of the competing memory contents (*modified hypothesis 4*). Instead of (complementary) weights one would have to assume competitive factors for such mutual inhibition.

There is an apparent advantage over the weight assignment. Competitive factor assignment implies that only odometer cues that are available from the start of a foraging trip contribute to navigation. In ants foraging in the dark [19], for example, any optic flow input that may occur later during travel is suppressed by the stride integrator memory that has been charged right from the outset. This would agree with the results of the 1–00, 1–01 and 1–11 experiments. The winner-takes-all scenario accounts for the fact that different (competing) home vectors, both global path integration vectors and local vectors (e.g. elicited by landmarks [20]), determine homing behaviour according to their vector strengths. For instance, in *Cataglyphis* ants close to the nest the path integration vector has little weight and may be overridden by a local vector elicited by nearby landmarks (e.g. [14, 17, 20, 32, 33]). Interestingly, this interpretation appears to be applicable to other navigating ant species. The Australian desert ant *Melophorus bagoti* inhabits visually cluttered environments and local landmark vectors are thus expected to assume higher relevance relative to path integration vectors. Indeed, *Melophorus* ants may be attracted to learned landmarks much earlier, that is, already with relatively large global path vectors, compared to *Cataglyphis* ants that live in visually barren landscapes [14, 34, 35]. Such species-specific differences may also affect the odometer reading where nest search behaviour is initiated.

In the winner-takes-all scenario, the critical 0–10 experiment is interpreted as follows. Both odometer memories are charged during outbound travel, as stated before. During the initial homebound travel with eye covers, the optic flow memory maintains its charge level and thus soon dominates stride integration memory. This accelerates depletion of the stride integrator memory and thus shortens homing distance. Subsequent removal of the eye covers re-activates homing driven by the maintained optic flow memory. During the ensuing (second) homebound travel only minimal charging of the stride integration memory occurs due to the higher charge level in the competing optic flow memory throughout homing.

Both of the above interpretations have to remain hypothetical at present. Only future experiments might clarify the situation, for example, by intercepting homing ants in a 1–0 paradigm. Removing the eye covers during the homebound travel, or during searching at a point distant from the search centre, might distinguish between additive and multiplicative interaction of the two odometer memories, for example.

### Scrutinising the hypotheses

The five hypotheses outlined in the Introduction feature prominently in the interpretations discussed above. How realistic are these assumptions, which of them are critical, and what arguments are there in terms of contradicting evidence and support?

**1. Separate odometer memories, charged in parallel**. This is not really a hypothesis since it has been demonstrated previously that the stride and optic flow odometers can work independently (1–11 experiment above, [9, 19]), and that the contents of the two memories cannot be transferred, at least not from the optic flow to the stride integrator memory [9, 36]. The reverse transfer, from stride integrator to optic flow memory, cannot be tested according to present knowledge; it would require the optic flow memory to drive homing without the stride integrator being active. The existence of separate odometer mechanisms and memories is not surprising. The stride integrator is associated with leg motor control and perhaps leg sensory feedback [22, 24, 31] and may thus be assumed to reside primarily in the thoracic ganglia. The optic flow odometer is associated with the eyes and optic lobes, where optic flow signals are evaluated, at least in dipterans [37, 38]. And although the nature and location of optic flow distance memories are unknown, the brain neuromeres would appear as likely structures. It is noteworthy in this context that the compass for skylight [39, 40] and landmark [41] orientation appear to be associated with the central complex, and with the protocerebral bridge in particular [39]. Strictly separate neuronal networks for the stride and optic flow odometer memories are not essential features, though, because separate memory functions may also be realised by a single network, as suggested for dung beetle orientation [40].

Parallel working of different orientation and navigation systems in desert ants–and elsewhere [42–44]—has frequently been implied, and recently it has been formally proposed [17,45]. In normal conditions, the parallel working of two odometers should not present any problems for the ants even if their relative weights vary with circumstances. After all, both odometers indicate the same distance, except in special behavioral situations. Such situations are, for example, the elimination of optic flow inputs by ventral eye caps (see also [7]) or the disabled stride integrator in carried ants [9].

**2. Interaction of the two odometers with different relative weights**. This hypothesis is suggested by the present experiments, although the exact mode of the interaction—for instance, additive, multiplicative, winner-takes-all—has to remain open. This has been discussed above in the context of interpreting the shortened homing distance in the 0–11 experiment and the second homing bout in the 0–10 experiment. Assuming a mode of interaction of the odometer memories along the lines presented above appears essential for explaining the present results. This is true together with the demands for separate odometer memories (1. above) and the initiation of a nest search as soon as the active distance memory has reached a value of zero (5. below). While odometer function and odometer memories are apparently independent for the stride and optic flow integrators [9, 19, 36], the two clearly interact in driving homebound travel. This result rules out completely independent superposition of the two odometer outputs. Incapacitating either odometer mechanism would not matter in the latter case because homing can be guided by either odometer alone across the correct homing distance—which is evidently not the case.

Let us first assume **additive weighting** of the two odometer memories in driving homebound travel. In that case, the weights for stride and optic flow odometers appear to be similar, about 0.6 and 0.4, respectively, during normal homing in experimental channels. This derives from the outcome of the 0-11-experiment, where ants—exclusively relying on stride integration during homing—start a nest search around 6 m (and hence 4 m must be attributed to the optic flow integrator).

In the next experimental step, the 0–10 trial, the nest search was centred close to 16 m, that is, about 10 m from the search centre in the 0–11 experiment and about 8 m from the release point after eye cap removal. With respect to the relative odometer weights, there are two ways of interpreting this result.

(i) One might assume that the already depleted stride integrator does not contribute to homing at all, perhaps due to optic flow appearing as the only reliable odometer with a depleted stride integrator. The relative weight of the optic flow memory should have shifted from 0.4 to 1.0, therefore. This agrees with the second search being centred on about 10 m from the release point of the 0–11 experiment, and on just above 8 m from the point of eye cover removal. The assumed contents of the optic flow memory at the point of eye cap removal is 10 m, after all.
(ii) Alternatively, the second search in the 0–10 experiment is initiated when stride and optic flow integrators are in balance. This assumes that both, stride and optic flow odometers are contributing to homing in the last part of the 0–10 experiment. The stride integrator drives the animal back into the direction of the first search centre since it now runs “backwards” (compare [20]), while the optic flow integrator drives the animal away from the first search centre according to the residual charge in the odometer memory. The search median measured from the second release point after removal of the eye covers was 8.15 m (“2. TP”, or 1. TP of second search). Considering the length of this second search of about 8 m and assuming an initial optic flow memory reading of 10 m, the relative weights of stride and optic flow integrators for the second search should be about 0.2 and 0.8, respectively. A weight of 0.8 still far exceeds the expected value of 0.4 calculated for the preceding 0–11 experiment. This would suggest adjustable weighting of the odometer memories according to context.

Assuming a (soft) **winner-takes-all** scenario, instead of the above additive weighting, may avoid the context-dependent “juggling” with adjustable weights. It will afford adjustment of the competition between the two odometer memories, however. The homing distance shortened to about 6 m in the 0–11 experiment would suggest that the two odometers do not really compete in an all-or-none fashion but in a multiplicative mode, for example. In the 0–11 experiment, the homing distance was shortened to about 6 m. To cover this result, depletion of the stride integration memory should have been accelerated by a factor of about 1.7 by the fully charged, competing optic flow memory.

When assuming that factor for the subsequent 0–10 experiment, the second homing bout after removal of the eye covers should have been about 6 m. This value is obtained when assuming a 10 m distance value in the optic flow memory and 0 m in the previously depleted stride integrator memory. During the second homing bout, the optic flow memory is depleted while the stride integration memory accumulates negative distance. After all, the ant moves in a direction opposite to home according to the stride integrator (see alternative (ii) above). The observed homing distance in the 0–10 experiment is just above 8 m, however. Some sort of non-multiplicative interaction of the two odometer memories might nonetheless satisfy the results of both experiments, 0–11 and 0–10.

Alternatively, one may consider 10.10 m homing distance obtained by comparing the search medians in the 0–11 and 0–10 experiments. This assumption implies that the optic flow memory completely dominates the stride integrator (see alternative (i) above), but not *vice versa*, as explained above.

In extreme situations, the relative contributions of the two odometer contents indeed assume an all-or-none mode, which is already implicated in hypothesis 1. That is, one odometer has a weight of 0 (e.g. the stride integrator in carried ants [9]) and the other, a weight of 1 (e.g. the optic flow integrator in carried ants when homing after loss of their carrier [9]). *Vice versa*, ants travelling with ventral eye caps during the outbound journey (1–11, 1–01, 1–00 experiments above) or at night [19] will rely on the stride integrator during homing and disregard the memory of the optic flow odometer that is empty.

In summary, regardless of whether assuming an additive weighting or a winner-takes-all scenario, the details of the interaction of the stride integration and optic flow memories have to remain open. Only future experiments may be able to clarify those details, as noted above. It is clear from the present results, however, that the two memories do interact in driving homebound travel. It further appears that the optic flow memory, always having a larger memory contents in the present set of critical experiments, tends to dominate stride integration memory to some extent. This is in contrast to previous studies that have examined the role of optic flow for distance measurement in ants [7, 23]. Distance memory contents were not manipulated in these experiments, however.

**3. Odometer memory is maintained with its current value if not active**. The maintenance of the distance component of a home vector over extended time periods (up to 4 days) has been demonstrated previously [46]. With the existence of two odometer memories it is to be expected that the two memory contents are maintained when the respective odometer is blocked, and that they can be charged and discharged separately. There is no current evidence for or against this hypothesis beyond the present set of experiments since this problem has not been addressed previously.

**4. Separate discharging of the two distance memories**. Much like stated above for hypothesis 1 (separate odometer memories, charged in parallel), this is not actually a hypothesis, but it has been presented since it forms an important premise of our preferred interpretations. Previous studies have demonstrated that the stride and optic flow odometers can work independently (1–11 experiment above, [9, 19, 36]), and that the two memories are thus charged and discharged independent of each other and are not transferable ([9], at least concerning transfer from optic flow to stride integration memory; above). As an important difference from the present study, one of the odometers was not functional from the outset in those previous experiments. The ants were either travelling in the dark or had their ventral eyes covered when leaving the nest. It is to be expected that an animal will rely only on the navigation cues available during the start of its journey and disregard cues that are unavailable. Even if the initially unavailable cues appear at a later time during the journey they should not be considered by a path integrator to avoid integration errors. This demand is actually realised in a rather straightforward way by a winner-takes-all interaction, as outlined above. This line of thought also applies to the 1-00- and 1-01-experiments, where the optic flow odometer is deprived of accumulating distance information during the outbound journey. It seems that the optic flow- integrator stays at zero during the homebound walk and does not contribute to the measurement of homing distance. Of course, these rather general statements would need further scrutiny for the different cue types, from the skylight compass with the tightly coupled elements sun position, polarisation pattern, and spectral gradient [3] to landmarks with or without familiarity [47].

**5. A nest search is initiated as soon as one of the odometer memories has been depleted, and the other odometer memory is either also zero, or the odometer is inactive and cannot be used; this holds although the second memory is still charged**. Desert ants are able to initiate a nest search in response to a number of different cues, depending on the situation. For example, a particular landmark array familiar to the ants from the nest surrounds is disregarded when encountered far from the nest. The landmark array will elicit a nest search, however, if the state of the path integrator indicates vicinity to the nest, even though not the nest position directly [20]. This fact can be used to lead homing ants away from their nest by presenting the landmark array right in front of them just before they reach the (fictive) nest position in a test field. The animals are made to run past and ever further away from the nest with repeated presentations of the landmarks [20]. The path integrator keeps constantly running in this situation, and once the animal is placed in an open test field without landmarks it truthfully returns to the (fictive) nest position. This demonstrates that several navigation mechanisms run in parallel in desert ants [3,14,17,45]. When put in conflict, it depends on the particular situation which cues are presently used to assess nest position and initiate a search. Often, a weak home vector is dominated by a strong vector [13, 32, 45]. Typically, short vector lengths are interpreted as weak vectors, among other reasons because a vector that has run down is also less definitive in its direction. However, landmarks may be disregarded initially but considered over the path integrator with experience if repeated disagreement occurs between the two cues at a feeding site [47].

It fits into this context that in the present experiments, the empty stride integrator is able to initiate a nest search when the optic flow integrator is blocked by ventral eye caps, even though the corresponding memory is apparently not empty (0-11-experiment). The odometers are part of the path integrator after all, although only its odometer component is examined in the present study for reasons of experimental feasibility.

### Conclusions and outlook

In summary, our experiments confirm previous studies demonstrating the independent operation of stride and optic flow odometers and the associated memories [7, 9]. More importantly, they demonstrate that stride and optic flow integrators interact dynamically in driving homing performance. The two odometer memories can interact with respective weights or according to a (soft) winner-takes-all mode, perhaps adjusted to the particular behavioral situation. It would appear necessary in future studies to decide between the different modes of interaction, such as additive or winner-takes-all, and to determine the weights or interaction factors assigned to the two odometer memories in different behavioral situations. Such behavioral situations are, for instance, meandering outbound and straight inbound travel, or substrates with different foothold qualities and optic contrast.

Our experiments were designed to examine just odometer mechanisms, while *Cataglyphis* ants do not assess travel distance *per se* but use it to calculate a home vector [48]. The far reaching independence of the stride and optic flow integrators may actually point towards the existence of two independent path integrators. Although in a different context, similar ideas have been put forward for the honey bee [48].

More generally, an understanding of the interaction of different odometer mechanisms appears valuable not just for animal navigation research but may inform discussions on sensor fusion in both behavioral contexts and potential technical applications [49–51]. After all, there is often more than one cue available for navigating animals (and humans, and navigation devices), and the navigator has to decide how to evaluate potentially conflicting information from these cues. In desert ant navigation, as suggested for a statistically optimal combination of compass and landmark cues [16,17], reliability of the two odometer mechanisms may contribute to the adjustment of the relative odometer weights or factors assumed above.

## Supporting information

S1 Fig Odometer performance in desert ants is influenced neither by eye painting nor by associated handling procedures. (A) Relative search density (RSD) plot of untreated control group 0–00 (n = 20). Ants performed inbound and outbound journeys without eye caps. After training, the ants were put into the test channel immediately and their homing and nest search behaviour was recorded. Relative search density (RSD) plot of 0-00-sham control group (n = 45) is shown here again for comparison (dashed green lines; compare Fig 2A). (B) Box-and-whisker plots and statistical analysis. There is no statistically significant difference between the 0–00 untreated control group and the 0–00 sham control group regarding both, 1. TPs (p = 0.403, t-test) and grand medians of search densities evaluated from 2.– 6. TPs (p = 0.261, t-test). Homing performance thus remained unaffected by the application of eye covers and associated handling.
(TIF)

S1 Table Raw data. Here you find the original raw data (turning point values) for all experiments as supplementary excel file. Listed are consecutive turning points (1.TP to 6.TP) of all tested individuals.
(XLSX)
